# Successful recognition and percutaneous coronary intervention of delayed severe multiple coronary artery dissection caused by cardiac surgery: a case report

**DOI:** 10.1186/s12893-019-0579-4

**Published:** 2019-08-23

**Authors:** Dengshen Zhang, Jun Shi, Jianglong Hou, Yingqiang Guo

**Affiliations:** Department of Cardiovascular Surgery, West China Hospital of Sichuan University, No.37 Guo Xue Alley, Chengdu, Sichuan 610041 People’s Republic of China

**Keywords:** Coronary artery dissection, Cardiac surgery, Percutaneous coronary intervention

## Abstract

**Background:**

Multiple coronary artery dissection is rare after cardiac surgery. It is difficult to recognize and is easily misdiagnosed as low output syndrome as a result of cardiopulmonary bypass (CPB).

**Case presentation:**

A 43-year-old woman who had undergone cardiac surgery presented with unstable hemodynamics, and progressively increasing lactate, B-type natriuretic peptide, and cardiac enzyme levels, along with electrocardiogram (ECG) changes. Angiography indicated the presence of severe multiple coronary artery dissection, and 3 stents were implanted, which improved the patient’s hemodynamic status and cardiac function.

**Conclusions:**

In the present report, we describe our experience with identifying and treating delayed severe multiple coronary artery dissection caused by cardiac surgery. Timely angiography is vital in patients suspected with coronary artery dissection, and percutaneous coronary intervention (PCI) should be considered as a treatment strategy for cases with severe multiple coronary artery dissection and unstable hemodynamics after cardiac surgery.

**Electronic supplementary material:**

The online version of this article (10.1186/s12893-019-0579-4) contains supplementary material, which is available to authorized users.

## Background

The prevalence of iatrogenic coronary artery dissection in the population is believed to be 0.07% [1]. Coronary artery dissection is usually caused during coronary angiography. Multiple coronary artery dissection rarely develops following cardiac surgery, and may result from the direct coronary ostia cannulation performed for cardioplegia solution delivery [2]. Most of these cases are identified during the surgical procedure and are promptly corrected. However, after surgery, coronary artery dissection may be difficult to recognize, and is easily misdiagnosed as low output syndrome and as a complication of cardiopulmonary bypass (CPB).

In the present report, we describe a case of successful detection of multiple coronary artery dissection through blood biochemical and electrocardiography (ECG) changes in a patient who had undergone cardiac surgery via transradial angiography, and was treated via implantation of 3 stents. This rare case highlights the importance of prompt detection via angiography in patients strongly suspected of having coronary artery dissection, as well as treatment via percutaneous coronary intervention (PCI) for those diagnosed with severe multiple coronary artery dissection with unstable hemodynamics after cardiac surgery.

## Case presentation

A 43-year-old woman without a history of coronary disease had undergone aortic/mitral valve replacement, tricuspid valvoplasty, and left atrial thrombectomy. Preoperative echocardiography indicated an ejection fraction (EF) of 0.5. Myocardial protection was performed through the antegrade (via the left and right coronary ostia) perfusion of cardioplegia solution during CPB. After aortic cross-clamp release was performed, normal prosthetic valve function was confirmed via transesophageal echocardiography (TEE). The CPB duration was 157 min. No complications were noted during the surgery, except for the additional time required to remove a substantial thrombus in the left atrium. The patient was successfully weaned from CPB, and was intubated and returned to the intensive care unit.

**Fig. 1:**
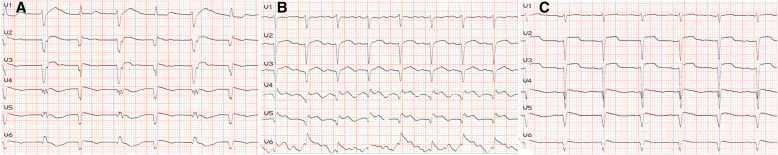
Changes on electrocardiography. **a** Frequent premature ventricular bigeminy. **b** Arched ST-segment elevation in V2–V6. **c** ST segment had descended

**Fig. 2:**
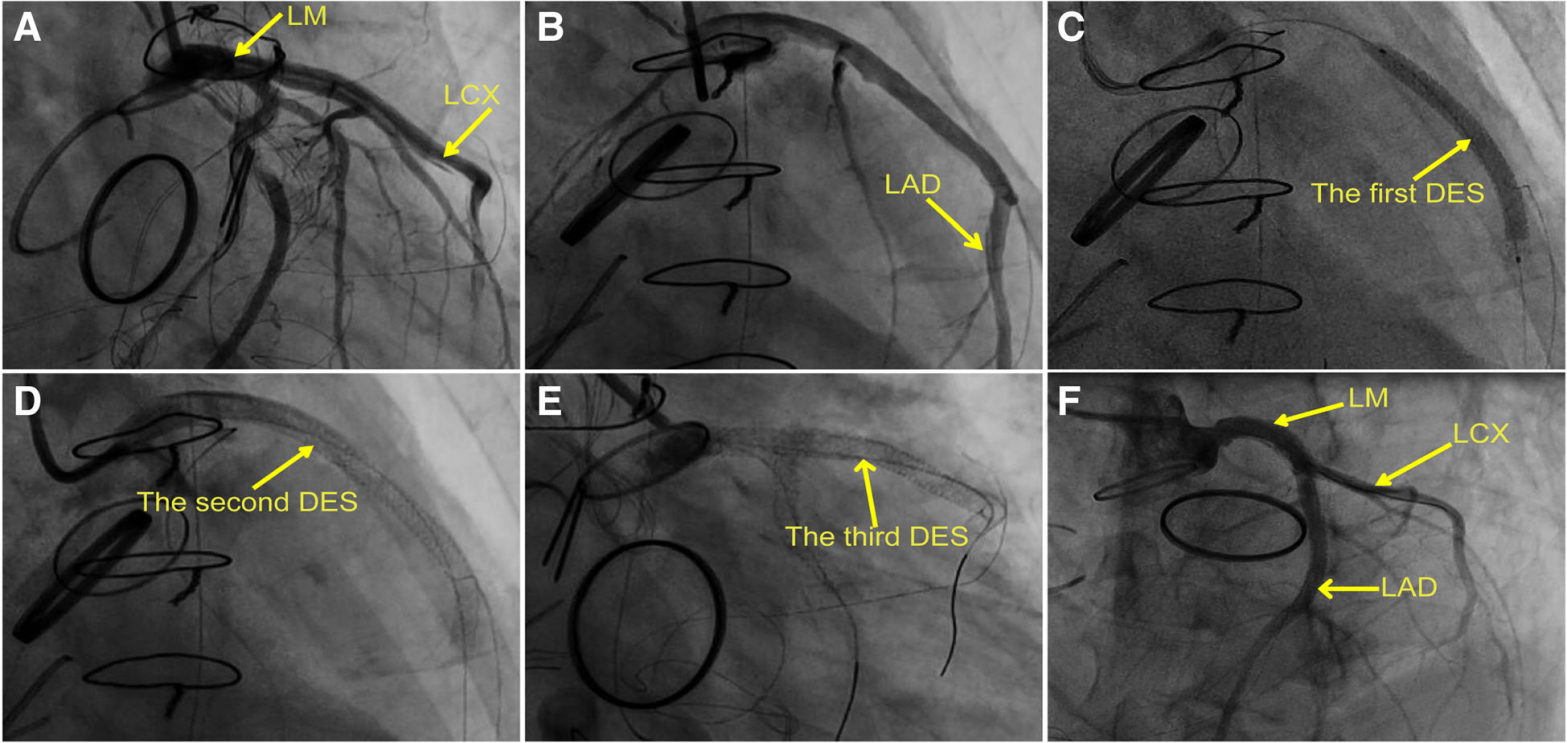
Angiography images of the multiple iatrogenic coronary artery dissection and the surgical procedure. **a** Coronary angiography showing marked dissection in the LM and LCX. **b** Coronary angiography showing marked dissection in the LAD. **c** Implantation of the first DES in the LAD. **d** Connection of the LM ostium with the LAD using a second DES. **e** Implantation of the third DES in the LCX. **f** Restoration of TIMI-III flow is confirmed in each branch. LM, left main coronary artery; LCX, circumflex artery; LAD, anterior descending artery; DES, drug eluting stent

Approximately 12 h after surgery, ECG indicated frequent premature ventricular bigeminy (Fig. 1a), whereas blood gas analysis indicated an increase in the lactate level to 10.2 mmol/L (reference value < 1.8 mmol/L) and uncorrectable metabolic acidosis. The patient’s serum type B natriuretic peptide (BNP) levels had also progressively increased. In addition, we observed the attenuation of the cardiac contraction amplitude via bedside echocardiography. After 14 h, the patient exhibited unstable hemodynamics, which required high doses of vasoactive drugs for blood pressure maintenance. Her adrenaline levels increased from 0.05 μg/kg/min to 0.1 μg/kg/min, whereas her norepinephrine levels increased from 0.03 μg/kg/min to 0.18 μg/kg/min. ECG indicated arched ST-segment elevation in leads V2–V6 (Fig. 1b). Bedside echocardiography indicated an EF of approximately 0.3 as well as wall motion abnormalities. Her serum troponin-T and creatinine kinase-MB levels had also increased to a peak of > 10,000 ng/L (reference value < 2.88 ng/L) and 300 μg/mL (reference value < 14 μg/mL), respectively, which indicated the presence of acute myocardial infarction. Coronary angiography indicated marked dissections in the left main coronary artery (LM), circumflex artery (LCX), and anterior descending artery (LAD) (Fig. 2a, b; Additional files 1 and 2). The patient successfully underwent PCI through radial access. The first drug-eluting stent (DES; 2.5 × 36 mm sirolimus-eluting stent; JW Medical Systems, China) was implanted in the LAD (Fig. 2c, Additional file 3), and a second DES (3.5 × 36 mm sirolimus-eluting stent) was implanted in the LM ostium (Fig. 2d, Additional file 4), thus sealing the dissection and recovering distal flow (Additional file 5). The LCX dissection was treated with a third DES (3.0 × 36 mm sirolimus-eluting stent) and distal flow was restored (Fig. 2e, Additional file 6). The restoration of TIMI-III flow was confirmed in each branch (the LM, LAD, and LCX; Fig. 2f, Additional file 7) and ST segment had descended (Fig. 1c). The patient recovered well and was followed up for 3 months after discharge. Echocardiography indicated that the EF significantly increased from 0.24 to 0.45.

## Discussion and conclusion

Coronary artery dissection is a rare complication of coronary intervention and cardiac surgery, with an incidence of approximately 0.07% [1]; thus, coronary artery dissection as a result of cardiac surgery is very rare. To our knowledge, only few reports have described the treatment of multiple coronary artery dissection after cardiac surgery through PCI [3]. Although the early recognition and treatment of coronary artery dissection are vital for patients, coronary artery dissection might often be overlooked after cardiac surgery. We believe that there are a few reasons for this occurrence. First, physicians rarely encounter coronary artery dissection. Second, patients do not complain of chest pain and other subjective symptoms due to their sedative status in the early postoperative period. Third, the condition can be easily misdiagnosed as low output syndrome as a result of CPB, which is associated with signs of cardiogenic shock caused by acute myocardial infarction. Surgeons may identify the unusual increase in myocardial enzyme levels, but these changes only occur > 12 h after surgery [4, 5]. Furthermore, the coronary artery dissection is dynamic and is usually type A or B, but might change to other serious types due to the impact of reduced blood flow.

In the present case, we believe that the occurrence of progressively increasing lactate and serum BNP levels, uncorrectable metabolic acidosis, wall motion abnormalities on bedside echocardiography, frequent premature ventricular bigeminy, and elevated ST-segment on ECG warrants closer attention and is vital for the early detection of acute myocardial infarction. Therefore, coronary artery dissection should be considered in patients who have undergone coronary perfusion via the coronary ostia. In fact, patients exhibiting these clinical features should promptly undergo emergent angiography, followed by PCI or coronary artery bypass grafting (CABG), depending on the patient’s status. For hemodynamically unstable lesions, rapid revascularization may be needed. During routine examination of the valve via TEE after operation, the esophageal probe is returned to the five-chamber view and adjusted by 30–45° to observe the coronary artery flap on the short axis of the aortic valve [6]; this view clearly shows the coronary artery dissection, and allows for prompt correction. Therefore, it is required to routinely observe the five-chamber view by TEE after cardiac surgery.

In the present case, although multiple operations, including aortic/mitral valve replacement and left atrial thrombectomy, were performed, no calcification of the aortic root was detected. However, as repeated perfusion was required for myocardial protection in this case, we believe an intimal tear at the ostium of the LM, LCX, and LAD may have caused the dissection. Therefore, surgeons should be careful and follow the standard protocol when delivering cardioplegia solution via the coronary ostia, and should avoid repeated intubations of the coronary ostia. In the present case, given the long-term effects of multiple coronary artery lesions, including dissection of the LM until the LAD, CABG should have been chosen instead [7, 8]. However, due to the progressive deterioration of the patient’s status and severely unstable hemodynamics, there was insufficient time available to perform CABG. Therefore, the patient was scheduled to undergo PCI, which increased her chance of survival, and led to improvements in her hemodynamic condition and cardiac function.

In conclusion, we have reported a case of successful recognition and treatment of delayed severe multiple coronary artery dissection caused by cardiac surgery. It indicates that timely angiography is vital in patients suspected with coronary artery dissection, and PCI should be considered as a treatment strategy for cases with severe multiple coronary artery dissection and unstable hemodynamics after cardiac surgery.

## Additional files

Additional file 1: Coronary angiography showing marked dissection in the LM and LCX. (AVI 8197 kb)

Additional file 2: Coronary angiography showing marked dissection in the LAD. (AVI 13573 kb)

Additional file 3: Implantation of the first DES in the LAD. (AVI 3432 kb)

Additional file 4: Connection of the LM ostium with the LAD using a second DES. (AVI 3392 kb)

Additional file 5: Recovery of distal LAD flow. (AVI 14085 kb)

Additional file 6: Recovery of distal LCX flow. (AVI 10245 kb)

Additional file 7: Restoration of TIMI-III flow is confirmed in each branch. (AVI 5637 kb)

## Data Availability

All data used and analyzed during this study are included within the article.

## Abbreviations

CABG: Coronary artery bypass grafting
CPB: Cardiopulmonary bypass
DES: Drug eluting stent
ECG: Electrocardiograph
LAD: Anterior descending artery
LCX: Circumflex artery
LM: Left main coronary artery
PCI: Percutaneous coronary intervention
